# Creativity and Emotional Exhaustion in Virtual Work Environments: The Ambiguous Role of Work Autonomy

**DOI:** 10.3390/ejihpe14070139

**Published:** 2024-07-19

**Authors:** Carlos Santiago-Torner

**Affiliations:** Faculty of Business and Communication Studies, Department of Economics and Business, University of Vic—Central University of Catalonia, 08500 Vic, Barcelona, Spain; carlos.santiago@uvic.cat

**Keywords:** creativity, emotional exhaustion, work autonomy, teleworking

## Abstract

The results regarding the relationship between creativity in virtual work environments and its influence on emotional exhaustion are inconclusive. Furthermore, autonomy, when it loses its original purpose of acting as a job resource, takes on an ambiguous role that needs further research. Objective: To analyze the relationship between creativity and emotional exhaustion, along with the role assumed by work autonomy in this link, in an online work context. Methodology: The sample was formed of 448 employees with university studies. The statistical analysis was conducted through a simple moderation process. Results: Creativity and work autonomy mitigate emotional exhaustion. In fact, work autonomy plays a moderating role regarding the relationship between creativity and emotional exhaustion. Control over work surely reduces the impact of work-related stressors, and this safety climate promotes adaptive and original responses that improve employees’ emotional health. However, when creative demands coincide with an autonomy that extends working hours, instead of establishing limits, this supposed benefit becomes a demand that prevents employees from disconnecting, until emotionally exhausting them. Conclusion: A virtual work environment is an ideal habitat for creativity and self-management to improve employees’ emotional health, as long as work autonomy acts as a resource.

## 1. Introduction

A virtual work environment is characterized by high flexibility in its design of tasks, allowing professional activities to be conducted from different places using communication instruments and networks [1]. This employment proposal provides a series of potential advantages, both for employees and for the different industrial sectors. It improves creativity [2] and attenuates emotional exhaustion (EE), among other things [3]. These positive aspects have been mainly attributed to the significant increase in autonomy, which allows redistributing functions with low interdependence [4]. However, it is also linked to drawbacks, e.g., a significant workload increase, which seems incoherent as its main objective is to improve the employees’ life quality [5].

The impact of teleworking has been addressed from different perspectives. However, there are still deep information gaps because many of the studies were conducted under certain demands related to unusual scenarios, specifically the COVID-19 crisis [3,6]. Furthermore, key aspects for organizational development, such as creativity, have only been interpreted in one direction, i.e., moderating or mediating variables that reduce its effect have been considered [7,8]. Therefore, this research was born with the objective of broadly determining the contextual effect of this work modality in one of the most important industries in the Colombian territory: the electricity industry.

The Colombian electricity sector is immersed in profound changes to respond to some of the worst economic scenarios of the last 25 years. High inflation and political uncertainty transfer more responsibility to this line of business, which also has an obvious social perspective [9,10]. In fact, after more than two years in which remote work has provided continuity to thousands of jobs, it is necessary to revise some factors that are vital for growth of the analyzed sector, especially creativity and work autonomy, as well as their effects on the employees’ emotional health.

Actually, innovation and the original ideas that stimulate it shape the Colombian electricity sector, which permanently needs a creative vision to progress and overcome the social and orographic difficulties of the national territory [11]. Furthermore, this energy industry is characterized for having an active population with high academic training, which is naturally linked to low interdependence and high flexibility. However, the articulation of these work components, and the possible impact on well-being, continue raising questions, particularly when this interaction occurs in a digital context.

Therefore, the main objective of this analysis is to evaluate the influence of creativity on EE in a virtual work environment, and determine if the moderation of work autonomy changes the direction of this possible link. Essentially, the impact of a workload requiring frequent creative contributions on EE has gone unnoticed by the scientific community. Only [12,13] have established some type of relationship between both constructs. On the other hand, the impact of work autonomy on EE has different interpretations. Recent studies support a positive association between autonomy and psychological well-being [14]. Likewise, Refs. [15,16] relate a low level of self-management with anxiety and EE. Nevertheless, other authors consider that excessive freedom to make decisions can also constitute an element of emotional risk [17,18].

This research is original and differs from other studies for various reasons. First, the possible relationship between creativity and EE has very few contrast studies, and even fewer in a virtual habitat. Second, the relationship between work autonomy and EE is inconsistent and requires a larger number of studies. Third, the different ranges of self-management and its moderating effect on the association between creativity and EE represent a new theoretical contribution that can help fill knowledge gaps.

### 1.1. Theoretical Framework

#### Creativity and Emotional Exhaustion (EE)

EE is a negative physical and mental reaction to permanent exposure to work-related stressors that overwhelm the employee’s resources [19,20]. Resources are aspects, contextual or personal, that help individuals counteract the possible demands of the job, and also contribute to the achievement of organizational goals. Without them, it is unlikely for the work activity to facilitate an adequate emotional well-being, or sufficient satisfaction, for it to be transformed into affection for the company and its needs [21]. Therefore, resource conservation is the key factor to prevent EE [22]. In this sense, a virtual work environment can be considered a resource due to its characteristics, e.g., flexibility, decreased distractors, low interdependence, and theoretical time savings [23]. Likewise, creativity and the personal traits that foster it, specifically openness to experience and extraversion, attenuate EE [12,24]. These two dimensions of personality coincide in a clear tendency to explore and engage in an active search for original ideas, which is possibly related to emotional well-being and affinity with the work activity [25].

Certainly, task complexity favors the creative capacity to solve a problem, and this difficult process includes ingenuity and interest to face a challenge responsibly, which positively affects physical and mental health [26]. Creativity is associated with a pattern of constructive behavior that tends to betterment through constant adjustments, which results in psychological safety and becomes a strength of humans [27,28]. Development of new solutions, perspectives, and even products surely represents a path towards psychological well-being and personal development [29]. Therefore, the following hypothesis is proposed: H1. Creativity is negatively related to emotional exhaustion in a virtual work environment.

### 1.2. Work Autonomy and Emotional Exhaustion (EE)

Work autonomy enables individuals to be able to organize most tasks voluntarily, to freely select the way to execute them, and to have certain independence in decision making [30]. Job position flexibility is a priority characteristic when structuring an employee’s functions, as it is closely associated to job satisfaction, psychological well-being, and motivation [14]. 

Returning to the JD-R model of resources and demands [21], work autonomy is a job resource that stimulates self-realization since it allows employees to take ownership of their achievements and deceptions. Furthermore, having the ability to decide improves the employee’s self-esteem by tolerating, providing solutions to, or taking advantage of the possible threats or opportunities that he or she has to face [31].

In fact, control over work moderates the relationship between work activity-related stressors (demands) and EE, as a possible risk of excess stress caused by an uninterrupted work overload. Consequently, work autonomy contributes to improving the employee’s emotional health [15].

Connecting concepts, Ref. [32] conclude that teleworking increases the satisfaction of the basic psychological needs of autonomy, competence, and relationship. Therefore, the employees’ confidence in their abilities to successfully face a complex situation, along with the ability to do so under strong self-determination and independence, directly impacts their satisfaction and reduces EE [33]. Autonomy is a relevant factor for employees’ work satisfaction and, when it is congruent with their skills, it is likely to increase their response system in the face of sustained and unexpected demands [34]. Consequently, the following hypothesis is proposed: H2. Work autonomy is negatively related to emotional exhaustion in a virtual work environment.

### 1.3. Work Autonomy Moderating Effect

Various studies mention that a virtual work environment favors a less interdependent and more specific design of work activities, which enriches employees’ autonomy in general [2]. However, the increasingly rapid development of new tools and information technologies forces workers to adapt and to expand their skills so they remain useful, which in turn increases mentally stressful job demands [35]. Additionally, qualitative activities, e.g., original and innovative ideas, are likely to influence the employees’ psychological well-being when coinciding with a high quantitative workload. Working under constant pressure requires a complementary emotional effort to deal with different work situations, which probably forces highly independent employees to exceed their working hours [36].

Control over a task remains a power that the employee has in order to decide the moment, place, and manner of undertaking a work activity. Indeed, the JD-R model suggests that job demands and control over them independently predict psychological well-being. It specifically sustains that a method able to establish limits attenuates the negative influence of job demands and weakens their impact on the employee’s emotional well-being [21].

In that sense, although most theoretical and empirical evidence proportionally relates work autonomy and well-being, a few studies establish that work autonomy that is too broad can intensify self-control in such a way as to imply adverse consequences on emotions. In other words, autonomy stops being a benefit and becomes a necessity due to the job characteristics. Therefore, it potentializes the action instead of restricting it [18].

Excessive autonomy can somehow be linked to unwanted job profiles subject to constant time pressure or little organizational support. Such context can absorb employees in such a way that they feel emotionally exhausted. In fact, continuous focus on a single direction leads to a certain instability in decision making, which establishes a curved pattern between autonomy and job satisfaction [31].

Work autonomy goes from being a resource to being a demand when the need to supervise, articulate, and design strategies becomes a requirement and causes permanent stress, especially in positions where decision making is an integral part of the job. In this case, work autonomy progresses by erasing the boundaries between work and life; that is to say, ample flexibility leads to greater dedication to the point that the organization paradoxically controls the employee’s life [37], through a culture of “long hours” that describes the detrimental effect of the supposed time freedom.

Virtual work environments have been consistently linked to excessive workloads due to an intensification of the workday [3]. In this sense, creative thinking uses a series of cognitive connections which, in addition to consuming a greater number of resources and energy, are also associated with extensive recovery [38]. Therefore, when continuous exposure to creative demands coincides with a work flexibility that does not establish limits and prevents disconnection, it is possible for employees to enter into a progression of instability tending to EE. Consequently, the following hypotheses are proposed: H3. Work autonomy positively moderates the relationship between creativity and emotional exhaustion in virtual work environments. H3.1: Creative demands, when moving through high work autonomy, tend to emotionally exhaust employees in virtual work environments.

## 2. Methodology

### 2.1. Type of Study

The research design was quantitative, transversal, non-experimental, and correlational. In accordance with [39], it represents an interaction between at least two variables, under a delimited and specific context.

### 2.2. Data Collection and Procedures

Data were collected for empirical tests from professional employees in the Colombian electricity sector who were teleworking. A total of 275 men and 173 women participated in the survey. A total of 13.40% (60) had staff under their charge, and 86.60% (388) did not. Job functions were distributed as follows: 4.50% (20) were managers; 8.90% (40) were in intermediate positions; 68.80 (308) were analysts; and 17.80% (80) were assistants. Organizational responsibility positions were distributed considering gender balance: 12% (21) women and 14% (39) men. The average age was 37 years (range from 20 to 69 years) and 82% (367) were under 50 years of age. Mean work experience was 10 years. All participants had studied at university and more than 60% (269) had graduate education. Distribution of online workdays was as follows: 25 (5.60%)—1 day; 95 (21.20%)—2 days; 98 (21.90%)—3 days; 103 (23%)—4 days; 127 (28.30)—5 days.

Sample data processing was conducted probabilistically by clusters with a confidence level of 95%. The STATS statistical program was used. The questionnaire was developed by a group of experts and sent to participants through Microsoft Forms online. The entire research was subject to an ethics committee at the end of 2021. The project was presented to the Colombian electricity sector in mid-2021, leveraging its community action meeting. Participating companies (6) were chosen in 2022, and the following documents were sent: voluntary consent and withdrawal, confidentiality agreements, data protection, and objectives presentation.

The time estimated to complete the survey was about thirty-five minutes, and processing was conducted on separate days. The main researcher presented the most important objectives of the research for about five minutes on each day. Additionally, he clarified the convenience of reading the questions carefully for answers to be reflective. Finally, the respondents’ rights were emphasized.

### 2.3. Instruments

The questionnaire questions were developed in Spanish as all the constructs have versions translated and accepted in this language. The study was conducted in a large part of the Colombian territory, specifically, in five different cities.

Control Variables: Based on previous studies [40], sex was used as a control variable. It was coded as 0 for men and 1 for women.

Creativity: Creativity was measured using the thirteen questions developed by [41]. Ten items belong to these authors, and three were adapted from the scale suggested by [42]. A six-point scale was used (1 = Strongly Agree; 6 = Strongly Disagree), e.g.: “I often have new and innovative ideas”. The Cronbach alpha obtained through the original scale was 0.96, using a five-point Likert scale. This research achieved a Cronbach alpha of 0.93. Employee’s creative traits in his or her job position were assessed as used by [43,44], with a Cronbach alpha of 0.93.

Work Autonomy: Work autonomy was measured using the three questions developed by [45]. The same prior six-point scale was used, e.g.: “I can decide on my own how to do my job”. The Cronbach alpha obtained by the original scale was 0.72. This research obtained a Cronbach alpha of 0.87. Employees’ control over the work that he or she is performing was evaluated, as used by [9,46], with a Cronbach alpha of 0.87.

Emotional Exhaustion: Emotional exhaustion was measured using the five questions developed by [47]. The same six-point scale was used, e.g.: “I am emotionally exhausted at my job”. The Cronbach alpha obtained through the original scale was 0.85, using a five-point Likert scale. This research achieved a Cronbach alpha of 0.90. The effect of the workload on individuals’ emotional resources was evaluated, as used by [48,49], with a Cronbach alpha between 0.85 and 0.90, respectively.

### 2.4. Data Analysis

The possible drawback of the Common Method Variation (CMV) is approached from different perspectives. Considering the guidelines of [50], Harman’s post hoc test of a single factor was used through the SPSS v.25 computer program. The extraction of factors specifies 32.312% of the total variance, and this percentage excludes the CMV problem for being less than the 50% limit. Furthermore, using six different sources of information, distributing the surveys on separate days, and using a separate questionnaire for each organization reinforces the idea that the CMV bias is not a significant problem for the data.

Calculated next were the descriptive statistics, and the various correlations between the three variables studied and the control variable (see Table 1). Subsequently, model relevance was established through two processes: (1). Confirmatory Factor Analysis (see Table 2); and (2). Convergent and discriminant validity (see Table 3). AMOS V.26 macro was used for these processes. Next, a multiple regression analysis was conducted using PROCESS V.3.5 macro, taking into account the considerations of [51]. The moderating role of the work autonomy variable was examined within the relationship built between creativity and emotional exhaustion. A simple moderation model (model 1) was used for this function with a Confidence Interval (CI) of 95% and 10,000 bootstrapping samples (see Table 4). To prevent multicollinearity problems, the mean centering method (creativity and work autonomy) was used. This technique mitigates the statistical drawbacks derived from an excessive correlation between predictor variables (greater than 0.90), and is helpful in a subsequent explanation of the factors part of the interaction [52].

**Figure 1:**
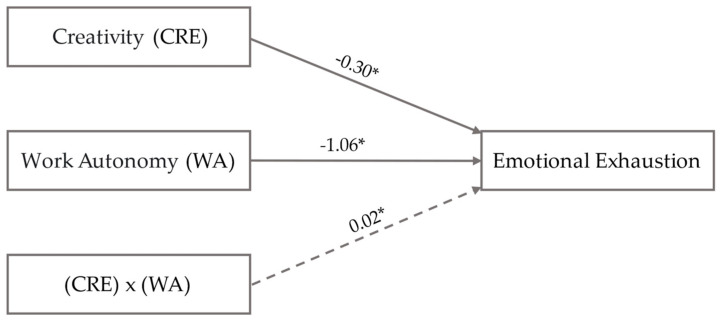
Moderation analysis results (unstandardized coefficients). Notes: The figure shows the proposed statistical diagram of simple moderation. CRE and WA significantly reduce emotional exhaustion. However, the combined effect of both variables changes the direction of the relationship. Significant correlations * (*p* < 0.05).

**Figure 2:**
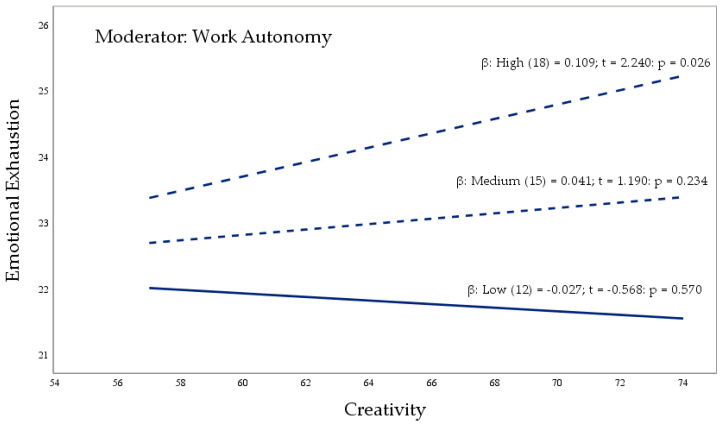
Work autonomy moderating effect. Notes: The figure shows the moderating effect of the work autonomy (WA) variable on the relationship between creativity (CRE) and emotional exhaustion (EE). CRE considerably limits the levels of EE through a low or medium perception of WA. On the other hand, CRE increases the sensation of EE with a high perception of WA.

**Figure 3:**
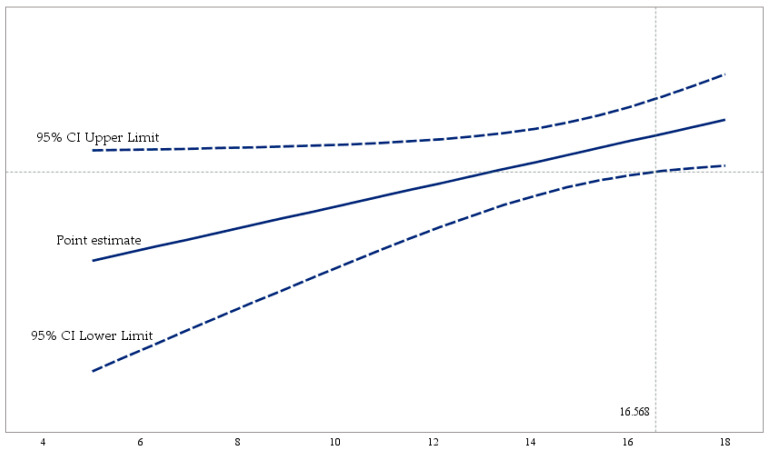
Creativity conditional effect—emotional exhaustion. Notes: The figure shows the region of statistical significance (upper right quadrant) where creativity (CRE), conditioned by the different values of the moderating variable work autonomy (WA), affects emotional exhaustion (EE). CRE does not attenuate EE when the perception of WA exceeds the limit of 16.568 (high).

Figure 1 shows the moderation model with unstandardized coefficients (AMOS V.26 macro). This analysis is adjusted using Table 4. Figure 2 (PROCESS V.3.5 macro) represents the moderation of work autonomy, with three conditional direct effects (low, medium, and high), on the relationship between creativity and emotional exhaustion. To conclude, Figure 3 (PROCESS V.3.5 macro) delimits the areas of statistical significance using the Johnson–Neyman technique, and the effect of the independent variable (creativity) on the dependent variable (emotional exhaustion), considering the values of the moderating variable (work autonomy).

## 3. Results

### 3.1. Reliability Analysis

The reliability of the three scales used in this study (see Table 1 and Table 2) is adequate. Cronbach’s alphas are above 0.70, which, based on [54], reveals reasonable acceptable uniformity. The analyses conducted indicate that there were no collinearity problems. Additionally, Table 1 indicates the number of items (N), the mean (M), the standard deviation (SD) and the different Pearson correlations. Creativity (CRE) is positively related to work autonomy (WA) (r = 0.304; *p* < 0.001), and negatively related to emotional exhaustion (EE) (r = −0.262; *p* < 0.001). Likewise, WA and EE are negatively related (r = −0.200; *p* < 0.001). Finally, the control variable sex does not have any significant links to the three main constructs, except for CRE.

### 3.2. Confirmatory Factor Analysis

The confirmatory factor analysis (CFA) was conducted using these absolute fit indices (Macro AMOS V.26): CMIN(χ^2^), likelihood ratio; (χ^2^/*df*), chi square with respect to the degrees of freedom; (RMSEA), root mean square error of approximation; (SRMSR), standardized root mean square residuals; (GFI), goodness-of-fit index. These values imply the degree to which the model can predict the matrix of observed covariances. Other incremental adjustment factors are used simultaneously: (IFI), incremental fit index; (CFI), comparative fit index; (NFI), normed fit index. These values verify the suggested model in relation to another model, which usually does not specify the link between constructs. The CFA confirms the validity of the proposed theoretical model.

### 3.3. Convergent and Discriminant Validity

Table 3 shows a second validation of the model through the validations suggested by [55]. The following analyses were conducted to verify the robustness of all the variables: (1). Cronbach’s alpha (2). Critical coefficients (CR). (3). Composite reliability (4). Average variance extracted (AVE). (5). Discriminant validity. (DV) Likewise, the critical coefficients (CR) were adjusted to the recommendations of [56] (>1.96; *p* value less than 0.05). The CFC and Cronbach alpha values were above 0.70, which guarantees the reliability of the constructs used. AVE factors were between 48% and 79%, which is significant. To have discriminant validity, the square root of AVE must be greater than the Pearson correlations between variables [57].

### 3.4. Validity Analysis

#### Hypothesis Tests

Figure 1 and Table 4 show the simple moderation analysis with non-standardized regression coefficients. Confidence Intervals (CI) are 95%, with 10,000 bootstrapping samples, and upper and lower vales (LLCI and ULCI) operate as dimensions. The regression analysis is irrelevant if 0 appears in the space delimited by the ranges. The coefficient of determination R^2^ contributes to understanding the relevance of the model used. In this case, it explains 21% of the dependent variable EE. The control variable sex is used to give consistency to the multiple combinations of the regression model.

Hypothesis 1 proposes that creative behavior is negatively related to emotional exhaustion. The linear regression justifies this relationship (β = −0.30, SE = 0.18, *p* < 0.05). Therefore, Hypothesis 1 is confirmed through route b1. Hypothesis 2 states that work autonomy is negatively related to emotional exhaustion. The linear effect b2 verifies this relationship (β = −1.06, SE = 0.16, *p* < 0.05) (see Figure 1 and Table 4).

Hypothesis 3 (see Figure 1 and Table 4), positive moderation of work autonomy in the relationship between creative behavior and emotional exhaustion, is verified by effect b3 (β = 0.023, SE = 0.048, *p* < 0.05). Hypothesis 3.1 (see Table 4) is tested using the low and medium conditional effects (β = −0.027, SE = 0.048, *p* > 0.05); (β = 0.041, SE = 0.034, *p* > 0.05) and high (β = 0.109, SE = 0.045, *p* < 0.05). Low or medium work autonomy does not influence the mitigating role of creative behavior on emotional exhaustion. However, the independent variable changes direction and positively influences emotional exhaustion when creative behavior and high work autonomy coincide.

Figure 2 graphically represents the interaction effect (moderation) of the work autonomy (WA) variable on the relationship established between creative behavior (CRE) and emotional exhaustion (EE). Using the pick-a-point method, PROCESS provides three different values for the moderating variable. These values are labeled as (1) low perception; (2) medium perception; and (3) high perception.

Figure 3 represents the conditional effect of creative behavior on emotional exhaustion for the different values adopted by the moderating variable (work autonomy). This graph defines the region of statistical significance, where the conditional effect studied between the X and Y variables is statistically significant for the different values of work autonomy (WA). It is observed that the effect of creative behavior on emotional exhaustion (represented by the line entitled Point Estimate) is statistically significant when WA has a score greater than 16.568, and ceases being significant with scores equal to or less than this value, with 16.80% of the sample above this value and 83.20% below.

## 4. Discussion

This research evaluates how creativity (X) can attenuate EE (Y) in a virtual work context. In this sense, work autonomy (WA) is used as a potential variable that can modify the intensity and orientation between X and Y. H1 confirms that creativity is negatively related to EE in virtual work environments, which partially agrees with the results obtained by [12,13]. Actually, the two previous studies were conducted in face-to-face contexts, and the latter failed to establish a significant relationship between creativity and EE.

On the other hand, hypothesis H1 does coincide with the findings of [58]. These authors consider that a virtual work environment is ideal for creativity to increase. Increased creativity depends on adequate cognitive absorption taking into account the use of information technology (IT). In that sense, cognitive absorption is described as a state of deep engagement that is associated with positive attitudes and a subjective experience that stimulates learning [59]. When a virtual worker copes with digital demands without apparent cognitive overload, he or she is able to be more creative through stable emotional states. That is, individual and contextual factors associated with IT buffer EE when resources, demands, and creative tensions are perfectly balanced.

A virtual work context is the right scenario in which to use non-traditional strategies that help solve the problems faced by the Colombian electricity sector, while making the most of employees’ skills. Creative capacity is surely intensified in spaces that allow more concentration on tasks and a high sense of control that prevents dependence on the environment [60]. Therefore, teleworking can be seen as a resource that, when properly managed, positively influences individual well-being. Therefore, it is possible that a work context that transmits well-being stimulates the creation of original ideas, and that in turn these are transformed into positive attitudes that attenuate EE [12].

In fact, creativity originates when cognitive abilities and individual traits find a suitable habitat, i.e., creativity is a response that a work environment with limited interaction and high flexibility can amplify [2]. In general, a work environment that promotes feelings of safety and trust is able to awaken persistence and enthusiasm in individuals to face complex jobs through new alternatives, stimulating employees to commit more and reducing burnout [13]. Emotional bonds with work and the organization surely imply greater effort and interest to maintain constructive points of view in the face of possible adversities or failures, along with a creative discovery, which limit the appearance of EE [25].

Actually, creativity and psychological well-being are actively related and strengthen each other. Creative thinking builds pathways that play a key role in self-development. Consequently, facing the challenges of a job position under a creative perspective, in addition to transferring meaning and value to the effort, improves individual skills and promotes a mood that favors the employee’s mental health [29].

The Colombian electricity sector can benefit from the mitigating relationship on the EE exerted by creativity in a virtual work environment from different points of view, e.g., by improving the worker’s virtual experience through richer and more attractive IT infrastructures. In this way, cognitive absorption will reach an optimal state and substantially improve creativity levels. Consequently, this context will further benefit EE reduction through intrinsically motivating experiences that bring about positive emotional states.

H2 confirms that work autonomy decreases EE, which coincides with [61]. These authors consider that work autonomy is a resource that allows an adequate reorganization of work, e.g., in rare environments such as virtual ones. In fact, Ref. [61] conclude, based on resource conservation theory, that work autonomy is a useful tool for acquiring other valuable resources. Thus, work autonomy tends to reduce certain harmful psychological manifestations, e.g., emotional exhaustion. To arrive at these results, Ref. [61] use SEM structural equation modeling through the statistical package Mplus version 6.12. Recent results such as those of [3] support the findings of [61]. In fact, Junça Silva and colleagues consider that the work autonomy associated with teleworking allows for better work schedule management that often leads to a better work–life balance. This effective self-management avoids work overload and EE. That is, telework is beneficial to employee mental health through effective concentration states that increase satisfaction and reduce certain stressors. These authors rely on resources and demands in the model JD-R proposed by [21] to justify their results.

In this sense, Ref. [14] concludes that task complexity associated with sufficient autonomy impacts the psychological well-being of the employee. Considering the JD-R model, work autonomy is a resource that allows individuals to improve self-confidence by taking ownership of the achievements and disappointments related to their job responsibility. This context of psychological safety facilitates better adaptation to the limitations and threats a work environment may present, which buffers a possible EE [31]. 

Autonomy and control over work can help employees be more effective regarding potential job demands, and reduce their negative results [18]. In fact, work autonomy effectively contributes to conservation of resources, which allows a better adaptation to contextual changes, preventing EE [61].

Likewise, a virtual work environment is a favorable scenario for employees to fully satisfy their psychological need for autonomy. Being autonomous implies a high degree of authenticity, which requires a strong integration between values and interests. In this sense, Refs. [32,33] establish a link between perceived autonomy and job satisfaction. Personal development and perception of achievement associated with job satisfaction prevent EE [62].

Finally, autonomy allows more capacity to self-regulate effort, interrupt the work activity, and facilitate recovery, which in turn preserves resources and reduces EE [34].

The Colombian electricity sector can accentuate the mitigating effect of work autonomy on EE in a virtual work environment in different ways, e.g., by ensuring a successful transition from face-to-face to virtual work. For this to happen, organizations must invest in procedures that foster the quality of relationships. In fact, trust in a virtual work environment is a critical factor that not only ensures the success of telework but also has a direct impact on the emotional health of the employee with high autonomy [3]. 

H3 and H3.1 determine the moderating role of work autonomy and, when this resource is increased inappropriately, it enables sustained creative demands to emotionally exhaust employees. This is perhaps the most important finding of this research.

Creative initiatives require a sufficient number of cognitive resources to thrive, along with emotional regulation linked to rest and disconnection [38]. When task complexity specifically coincides with high pressure on time for the task to be resolved, employees are likely to use more autonomy to expand their effort and dedication to the work activity [11]. Therefore, flexibility goes from being a positive process to being an obligation, and becomes a control mechanism that exhausts the employee [18]. Undoubtedly, when a work resource loses its primary meaning, it becomes ambiguous and may change its direction until becoming a demand that negatively influences motivation, well-being, and balance between work and home [31].

In general, excess work autonomy also tends to lead to personal objectives with a higher scope, which paradoxically exercise control over the employee’s lifestyle, limiting it. This tension is particularly evident in teleworking [37]. These authors develop the autonomy–control paradox. High work autonomy, instead of becoming a resource, is transformed into greater control and intentionality towards the work activity. That is, it becomes a demand and a strong constraint. Therefore, high labor flexibility can prevent the employee from having a good capacity to adapt and the tensions between work and life tend to compete with each other, affecting their emotional stability. In this sense, the Colombian electricity sector has the possibility of establishing work climates that promote flexible policies through less rigid rules. In fact, it is hardly feasible to encourage a high level of work autonomy through dissuasive policies that punish its use. In addition, disconnection policies in a virtual environment can prevent employees from choosing extended working hours that substantially increase stress levels and are detrimental to their emotional health. An organizational culture that values employee well-being will seek to alleviate tensions through sustainable workplace design that is geared toward improving the employee’s life outside of work.

In that direction, Ref. [30] analyzed the theoretical protective role of work autonomy in a virtual work environment, concluding that the effect of job flexibility on EE depends on personal tendencies, i.e., poor management of life and work boundaries turns work autonomy into a reason to intensify work activity, and the need for increased autonomy becomes a precursor to excessive work dependence resulting in EE.

## 5. Conclusions

A virtual context allows the limits of the job position to be actively shaped and the tasks to be redefined. Actually, this continuous and dynamic process is a reflection of the individual creative condition. In this sense, tactics seeking balance between challenging demands and sufficient resources to successfully address them give rise to positive psychological reactions that prevent burnout and job dissatisfaction. Certainly, capacity for adaptation and a decreasing restructuring of demands, which hinder creative behavior, prevent sustained exposure to stressful contexts. These are contexts that tend to overwhelm the employees’ resources until exhausting their emotional options. In conclusion, creativity is by itself a work resource that, in a flexible and equitable work environment with a limited number of distractions, turns into a personal development tool that prevents EE.

Autonomy and control over work activity are presented as resources that help employees to effectively cope with work-related demands and reduce their negative effects. Furthermore, an adequate self-management capacity enables EE to be consistently limited during organizational change. In this direction, a virtual work environment is a poorly centralized option that requires certain emotional transitions. Therefore, appropriate work flexibility is likely to prevent the stress and psychological distress that often precede, or are linked to, EE.

Finally, when employees are not able to reduce certain work demands and creative demands remain high, it is likely for them to use the scope of work autonomy as an adaptive strategy. In other words, they seek to release energy and work resources by extending their work day. Specifically, in the face of time pressure and task complexity, they use a simultaneous resource that theoretically offers the possibility of adjusting the work itinerary.

However, control over work only buffers the adverse repercussions of high pressure when it is truly applicable to the nature of the demand. When this strategy is erroneous, it implies a constant mismatch between advantages and disadvantages. Instead of allocating resources, it acts in the opposite way, deteriorating them, until reaching EE. In fact, creativity thrives in a work environment that offers the right resources, i.e., sufficient disconnection and cognitive rest. When these characteristics disappear, the primary role of autonomy also disappears and coping responses become harmful. That is, control passes from the employee to the job position and this situation usually results in EE.

### Limitations and Future Research

This research has an important weakness: the conditional process model, being subject to cross-sectional design, cannot draw exact conclusions about the causes. However, some limitations evidenced in other studies, for instance the influence of the common method variance, could be attenuated. This drawback occurs when collecting the different variables, whether dependent or independent, through a single source. Partially following the indications of [50], six reporting sources were used. Additionally, to exercise some control over the contextual effect, surveys were distributed on different days and the order of the questions was changed as each organization had its own questionnaire. Measuring the dependent and independent variables at different times was not possible. Furthermore, two independent procedures are used to minimize the social desirability bias: (1) surveys were completely anonymous, and (2) the importance of responding appropriately was emphasized in previous meetings.

Regarding future research, there are a number of moderation mechanisms that may also explain the relationship between creative behavior and EE, e.g., emotional commitment or perceived social support. It is also possible to verify if different contexts or climates can be situational aspects that explain the causal relationship between creative behavior and EE; specifically, if ethical climates—benevolent, principled, or self-interest—and even an ethical leadership style, can function as significant resources to mitigate EE.

## Figures and Tables

**Table 1:** Descriptive statistics.

Variables	N	M	SD	Sex	CRE	LA	EE
Sex	1	0.39	0.488	x			
Creativity (CRE)	13	64.89	7.92	−0.189 *	0.690		
Work Autonomy (WA)	3	14.91	2.50	−0.039	0.304 *	0.890	
Emotional Exhaustion (EE)	5	23.11	5.55	0.028	−0.262 *	−0.200 *	0.810

Notes: The table shows the calculation of the descriptive information and the Pearson correlations. Discriminant validity (diagonal) is the square root of average variance extracted. (N) Number of items. (M) Mean. (SD) Standard deviation. Significant correlations * (*p* < 0.05). CI (95%) (n = 448).

**Table 2:** Confirmatory Factor Analysis (CFA).

Goodness-of-Fit Measure	Acceptable Fit Levels	Results
CMIN (χ^2^)	χ^2^ (small)	465.24
χ^2^/df	<3	2.60
RMSEA	<0.06	0.046
SRMSR	<0.08	0.066
GFI	>0.90	0.916
IFI	>0.90	0.922
CFI	>0.90	0.914
NFI	>0.90	0.911

Notes: The correct fit of the model is usually influenced by different causes (1). Number of factors. (2). Number of response points. (3). Sample size. The adequate fit shown in the table is probably due to the low number of factors analyzed (3), to the high number of response points (6), and to the sample size (n = 448). In fact, samples close to 450 people have a 76.7% probability of fitting [53].

**Table 3:** Convergent and discriminant validity.

	ALPHA ^1^	CR ^2^	CFC ^3^	AVE ^4^	DV ^5^
CRE	0.93	>1.96	0.820	0.480	0.690
WA	0.87	>1.96	0.850	0.790	0.890
EE	0.90	>1.96	0.860	0.650	0.810

Notes: The table shows the degree to which the measure of the items that include the same concept are correlated (convergent validity), and the theoretical difference between the different constructs (discriminant validity). ^1^ Cronbach’s alpha. ^2^ Critical coefficients. ^3^ Composite reliability. ^4^ Average variance extracted. ^5^ Discriminant validity.

**Table 4:** Moderation analysis.

Effect		Route		*p*	t	SE	LLCI	ULCI
Effect CRE ^1^—EE ^2^		b1	−0.299	0.001	−5.362	0.181	−0.638	−0.112
Effect WA ^3^—EE		b2	−1.064	0.001	−5.245	0.162	−2.513	−0.385
Effect CRE × WA—EE		b3	0.023	0.030	2.859	0.048	0.001	0.045
Effect Sex Control Variable			0.155	0.768	0.296	0.525	−0.876	1.186
Ef. Condition WA (CRE-EE)	Low (12)		−0.027	0.570	−0.568	0.048	−0.121	0.067
	Medium (15)		0.041	0.234	1.191	0.034	−0.027	0.108
	High (18)		0.109	0.026	2.239	0.045	0.013	0.204

Notes: The table shows the effect of ^1^ creativity on ^2^ emotional exhaustion; the effect of ^3^ work autonomy on EE. The combined effect of CRE and LA (moderation) on EE. The different conditional effects of the moderating variable LA (low, medium and high). (R^2^ = 0.215). Statistical strength (f^2^) is medium (0.240).

## Data Availability

The original data presented in the study and the used questionnaire are openly available in The Open Science Framework repository at https://osf.io/w2g5b/?view_only=f8b9995262ed469eab5413f302dd83c4, accessed on 5 June 2024.
